# Physical fitness in Austrian elementary school children prior to and post-COVID-19

**DOI:** 10.3934/publichealth.2023034

**Published:** 2023-05-26

**Authors:** Clemens Drenowatz, Gerson Ferrari, Klaus Greier, Sitong Chen, Franz Hinterkörner

**Affiliations:** 1 Division of Sport, Physical Activity and Health, University of Education Upper Austria, 4020 Linz, Austria; 2 Escuela de Ciencias de la Actividad Física, el Deporte y la Salud, Universidad de Santiago de Chile (USACH) Santiago 7500618, Chile; 3 Department of Sport Science, University of Innsbruck, 6020 Innsbruck, Austria; 4 Division of Physical Education, Private Educational College (KPH-ES) 6422 Stams, Austria; 5 Institute for Health and Sport, Victoria University, Melbourne 8001, Australia; 6 Olympic Training Center Upper Austria, 4020 Linz, Austria

**Keywords:** body weight, cardiorespiratory endurance, muscular strength, muscular power, speed, agility, flexibility, motor competence

## Abstract

**Background:**

With the emergence of the COVID-19 pandemic, many countries implemented policies that included movement restrictions, social distancing and school closures in order to control the spread of the virus. Even though these actions may have been necessary to save lives, there have been some unintended consequences that could affect future public health.

**Methods:**

The present study uses data from more than 24,500 Austrian elementary school children (51.2% male) that participated in a state-wide fitness evaluation program, which was initiated in the 2016/17 school year. In addition to body weight and height, data on cardiorespiratory endurance, muscular power, speed, agility, flexibility and object control were collected from three cohorts prior to the implementation of movement restrictions (school years: 2016/17, 2017/18, 2018/19) and one cohort in 2022, after the majority of COVID-19 policies had been lifted.

**Results:**

Body mass index percentiles were significantly higher in children post-COVID-19 (p < 0.01). Further, cardiorespiratory endurance, agility and flexibility were significantly lower post-COVID-19 compared to the years preceding movement restrictions (p ≤ 0.01), while absolute muscular strength was higher in the year 2022 (p < 0.01).

**Conclusion:**

Given the detrimental effects of COVID-19 policies on physical fitness in children, additional efforts are necessary that include versatile opportunities for physical activity and the promotion of physical fitness in order to modify the observed negative health trajectories and ensure future public health.

## 1. Introduction

The benefits of physical fitness on the general development and health of children have been well documented [1],[2]. Both cardiorespiratory and muscular fitness are associated with a reduced risk for obesity and cardiometabolic diseases in children [3]–[5]. In addition, there are beneficial associations of various components of physical fitness with health-related quality of life [6],[7], academic achievement [8],[9] and cognitive function [10],[11]. It is also a critical component for the promotion of an active lifestyle, as physical fitness has been defined as a set of attributes that allows individuals to perform activities of daily living without undue fatigue and with ample energy for emergencies or leisure-time activities [12]. High levels of physical fitness during childhood, therefore, can decrease health problems from an early age and positively affect future health [13]. Nevertheless, there has been a decline in physical fitness in youth over the last several decades, which has been mirrored by the adult population in their accompanying detrimental health effects, such as increased risk for cardiovascular and metabolic disease, various types of cancer and all-cause mortality [14],[15].

Even though physical fitness is influenced by genes and the environment [1], low physical fitness levels in youth have been predominantly attributed to behavioral changes such as a decline in physical activity (PA), along with an increase in screen time [16]–[18]. These behavioral changes also contributed to a high prevalence of overweight and obesity [19]–[21], which is related to physical fitness as well [22],[23]. On top of this pandemic of physical inactivity [24], the coronavirus disease 2019 (COVID-19) was declared as a global pandemic by the World Health Organization [25]. In order to contain the spread of the virus, various policies were implemented, including stay-at-home advisories, social distancing and the closure of schools and sports facilities, which affected a large number of children and adolescents across the globe [26]. Even though these measures were implemented to save lives [27], there have been unforeseen consequences due to the alteration of daily routines and various behaviors. Across the globe, daily steps, for example, declined by 27.3% during the first 30 days of movement restrictions [28], while the screen time of children and adolescents increased by 52% [29]. In Austria, the first nationwide lockdown was initiated in March 2020, which prohibited people from leaving their homes except for covering their basic needs of daily life, providing help for family members, physical and psychological recreation and life-threatening situations. Elementary schools remained closed for 2 months during this initial lockdown and, subsequently, only half of the students were allowed in the classroom. Due to rising COVID-19 cases in the fall, schools were forced to return to distance learning again in November of 2020 and at various times in the following years [30]. Accordingly, homeschooling and social distancing became a normal part of life. Even though some of the early movement restrictions had been lifted at later stages of the pandemic, there remained barriers to engaging in sports and exercise in clubs or group settings, as well as physical education at schools, for more than two years.

The detrimental physiological and behavioral consequences of the COVID-19 pandemic, even in people who were not diagnosed with the COVID-19 virus, have been well documented [31],[32]. Despite the fact that children were less vulnerable to adverse outcomes from a COVID-19 infection, they did experience significant changes to their daily routines, which affected their health behaviors [33]–[35]. Given the limited access to various facilities, previous studies showed a decline in time spent in organized sports and total PA in children and adolescents while screen time increased [34],[36]–[40]. A German study, on the other hand, showed that habitual PA levels increased during the first lockdown [35]. These changes, however, did not last until later in the pandemic [41].

These behavioral changes were also associated with significant weight gain in children [42]–[44], as well as a decline in cardiorespiratory fitness, while effects on muscular strength were less consistent [45]–[47]. Most studies, however, only looked at a single time point prior to and shortly after COVID-19 restrictions and, therefore, may not be able to differentiate between a secular trend of declining fitness levels and the long-term consequences of COVID-19 policies in youth. The present study used data from a state-wide fitness testing program that started in 2016 and, therefore, allowed us to explore differences in the development of physical fitness in elementary school children over a 3-year time span prior to COVID-19 restrictions and the 2 years under various movement restrictions that had been implemented to mitigate the spread of the virus.

## 2. Materials and methods

In the 2016/17 school year, the state-wide project “Wie fit bist du?” (i.e., How fit are you?) was established. It consists of annual fitness tests in Upper Austrian elementary schools and has been described in detail previously [48]. All elementary schools in the federal state of Upper Austria received information about the project and were invited to participate. Study procedures were in accordance with the 2008 Declaration of Helsinki, and the study protocol was approved by the Upper Austrian School Board. Parents provided written informed consent prior to data collection and children provided oral assent at the time of measurement.

Data collection occurred during a single visit at the participating schools throughout the academic years. Until the end of the 2018/19 school year, physical fitness assessments were performed in more than 200 schools, resulting in a sample of 18,168 (51.3% male) children between 6 and 11 years of age, with valid data prior to the implementation of COVID-19 restrictions. Since the restart of the project in spring 2022, i.e., after restrictions had been lifted in schools, fitness assessments have been carried out in 168 schools, resulting in a sample of 6403 (50.7% male) children.

All measurements were carried out by trained technicians during a single session lasting between 90 and 120 minutes per group. Anthropometric measurements were taken according to standard procedures, with children in gym clothes and being barefoot. Body weight was measured with a portable electronic scale (Seca 878 dr, Seca, Hamburg, Germany) to the nearest 0.1 kg, and height was measured with a portable stadiometer (SECA 2013, Seca, Hamburg, Germany) to the nearest 0.5 cm. Body mass index (BMI; kg/m^2^) was calculated and converted to BMI percentiles (BMIPCT) using German reference values (49). Children with a BMIPCT above the 90^th^ and 97^th^ percentiles were classified as overweight and obese, respectively, while those below the 10^th^ percentile were classified as underweight.

Following the anthropometric measurements, children completed eight physical fitness tests to assess muscular power, speed and agility, cardiorespiratory endurance, flexibility and object control. Specifically, participants performed a countermovement jump on a force plate and a medicine ball push to determine relative and absolute muscular power, respectively. Speed was assessed via a 10-m sprint and a 6-second tapping test. A standardized obstacle course that required a forward role, jumping over and crawling under obstacles, as well as directional changes, was used to determine agility. Cardiorespiratory fitness was assessed with a 6-minute run, and flexibility was assessed with a stand-and-reach test. A 30-second throw-and-catch task with a European handball (size 1) was used to assess object control. Except for the countermovement jump (three attempts) and the 6-minute run (one attempt), each test was performed twice with sufficient recovery time between attempts. In addition to raw performance scores, z-scores were calculated using the total sample as the reference group. The best results were used in the analyses. Fitness tests were administered in random order, except for the 6-minute run, which was completed at the end of the testing session.

*Statistical analyses*. Descriptive statistics are reported as prevalence or mean with standard deviation. Differences in anthropometric characteristics across assessment years were initially examined via ANOVA and Bonferroni adjustment for post-hoc analyses, while MANOVA was used to examine differences in physical fitness. Given the age differences across study cohorts, MANCOVA, adjusting for age, was subsequently used to examine differences in BMIPCT and physical fitness across the total sample, and separately for boys and girls. In a second model, BMIPCT was included as an additional covariate in order to account for the potential impact of body weight on physical fitness. All statistical analyses were performed with IBM SPSS Statistics 28.0 (SPSS Inc., IBM Corp., Armonk, New York, NY, USA), with the level of significance set at p < 0.05.

## 3. Results

Physical fitness was assessed in a total of 24,571 (51.2% male) elementary school children (age: 8.4 ± 0.8 years) between the years of 2016 and 2022. Table 1 provides an overview of the sample sizes prior to COVID-19 movement restrictions (2016/17 to 2018/19 school years) and the number of children measured in 2022 post-COVID-19 movement restrictions. There was no significant difference in sex distribution between measurement years (chi-square = 2.05, p = 0.56).

**Table 1.** Number of participants in the respective school years.

	2016/17	2017/18	2018/19	2022
Total Number	6377	6077	5714	6403
Girls	3102 (48.6%)	2989 (49.2%)	2751 (48.1%)	3157 (49.3%)
Boys	3275 (51.4%)	3088 (50.8%)	2963 (51.9%)	3246 (50.7%)

Children participating in 2022 were significantly younger than participants in the 2016/17 and 2018/19 school years (p ≤ 0.01), but they were significantly older than the participants in 2017/18 (p < 0.01) (Table 2). There were also significant differences in anthropometric characteristics (p < 0.01). Specifically, children assessed in 2022 were taller and heavier compared to those assessed prior to the implementation of COVID-19 policies. While there was no significant difference in BMIPCT between the 3 years prior to COVID-19, BMIPCT was significantly higher in 2022 compared to all of the previous measurement years (p < 0.01). Sex-specific results also showed significantly higher BMIPCT in girls in 2022 compared to pre-COVID-19 years (p < 0.01), while the differences were less pronounced in boys. In fact, BMIPCT did not differ significantly between 2018/19 and 2022 in boys.

**Table 2.** Age and anthropometric characteristics in the respective school years for the total sample, and separately for boys and girls.

	2016/17	2017/18	2018/19	2022
Age (years)^1,2,3^	8.5 ± 0.8	8.3 ± 0.7	8.4 ± 0.8	8.3 ± 0.8
Boys only^1^	8.5 ± 0.8	8.3 ± 0.8	8.4 ± 0.8	8.4 ± 0.8
Girls only^1,2^	8.4 ± 0.8	8.2 ± 0.7	8.4 ± 0.8	8.3 ± 0.8
Body height (cm)^1,2,3^	132.7 ± 7.1	131.8 ± 6.8	132.1 ± 7.2	133.6 ± 7.4
Boys only^1,2,3^	133.4 ± 7.0	132.6 ± 6.8	132.7 ± 7.2	134.3 ± 7.2
Girls only^1,2,3^	131.9 ± 7.1	130.9 ± 6.6	131.4 ± 7.1	132.9 ± 7.5
Body weight (kg)^1,2,3^	30.0 ± 7.1	29.5 ± 7.0	29.8 ± 7.2	31.0 ± 7.9
Boys only^1,2,3^	30.4 ± 7.1	30.1 ± 7.2	30.3 ± 7.2	31.4 ± 7.9
Girls only^1,2,3^	29.5 ± 7.0	28.9 ± 6.8	29.4 ± 7.2	30.6 ± 7.8
BMI percentile^1,2,3^	51.3 ± 29.8	51.8 ± 29.7	52.4 ± 29.7	54.8 ± 30.2
Boys only^1,2^	52.0 ± 29.4	53.0 ± 29.4	53.8 ± 29.3	55.2 ± 29.8
Girls only^1,2,3^	50.6 ± 30.3	50.5 ± 29.9	51.0 ± 30.0	54.3 ± 30.6

*Note: Values are mean ± SD. ^1^2022 sig. different from 2016/17 (p < 0.01); ^2^2022 sig. different from 2017/18 (p < 0.01); ^3^2022 sig. different from 2018/19 (p < 0.01).

**Table 3.** Prevalence of underweight, healthy weight, overweight and obesity in the respective school years for the total sample, and separately for boys and girls.

	2016/17	2017/18	2018/19	2022
Underweight (%)	9.1	8.3	8.0	7.6
Boys only	7.9	7.4	6.9	6.7
Girls only	10.3	9.4	9.1	8.5
Healthy body weight (%)	76.7	77.0	77.0	74.4
Boys only	77.5	77.1	77.3	75.1
Girls only	75.8	76.9	76.6	73.6
Overweight (%)	8.3	8.2	8.7	9.6
Boys only	8.6	8.6	8.9	9.9
Girls only	8.0	7.8	8.4	9.4
Obese (%)	6.0	6.4	6.4	8.5
Boys only	6.1	6.9	6.9	8.4
Girls only	5.9	6.0	6.0	8.6

In line with the changes observed in BMIPCT, the prevalence of overweight/obesity increased from 14.3% in 2016/17 to 18.1% in 2022 (p for trend < 0.01) (Table 3). Over the 3 years prior to COVID-19, the prevalence of overweight/obesity increased by only 0.8% (from 14.3% to 15.1%), while there was a 3% increase from 2018/19 to 2022 when movement restrictions and social distancing policies were implemented. Even though the prevalence of being underweight was higher in girls compared to boys at all time points, the trends in the development of overweight/obesity did not differ in the sex-specific analyses.

**Table 4.** Components of physical fitness in the respective school years for the total sample, and separately for boys and girls.

	2016/17	2017/18	2018/19	2022
Countermovement jump (cm)^1,2,3^	19.9 ± 3.8	19.8 ± 3.8	19.9 ± 3.9	19.5 ± 3.9
Boys only	20.4 ± 3.8	20.3 ± 3.9	20.4 ± 4.0	20.1 ± 4.0
Girls only^1,2,3^	19.4 ± 3.4	19.3 ± 3.6	19.4 ± 3.7	18.9 ± 3.7
Medicine ball (cm)^1,2,3^	353.2 ± 74.1	346.2 ± 72.3	352.1 ± 77.3	356.7 ± 77.7
Boys only^1,2,3^	376.6 ± 73.1	371.1 ± 70.8	374.6 ± 78.9	379.2 ± 76.8
Girls only^1,2,3^	328.5 ± 66.7	320.4 ± 62.0	327.8 ± 67.6	333.4 ± 71.5
Tapping (# / 6 s)^2^	45.4 ± 7.5	44.5 ± 7.3	45.0 ± 7.7	45.0 ± 7.7
Boys only^2^	47.1 ± 7.3	46.4 ± 7.0	47.0 ± 7.2	47.3 ± 7.6
Girls only^1^	43.7 ± 7.3	42.5 ± 7.1	42.9 ± 7.7	42.6 ± 7.7
10m sprint (s)	2.27 ± 0.16	2.29 + 0.17	2.28 ± 0.18	2.28 ± 0.19
Boys only	2.24 ± 0.16	2.25 ± 0.17	2.25 ± 0.17	2.25 ± 0.18
Girls only^1^	2.29 ± 0.16	2.32 ± 0.17	2.32 ± 0.19	2.32 ± 0.19
Agility run (s)^1,2,3^	19.6 ± 3.4	20.2 ± 3.7	20.1 ± 3.7	20.7 ± 5.3
Boys only^1,2,3^	19.2 ± 3.5	19.7 ± 3.7	19.6 ± 3.8	20.1 ± 4.5
Girls only^1,2,3^	20.1 ± 3.2	20.8 ± 3.7	20.6 ± 3.6	21.3 ± 5.9
Throw & catch (# / 30 s)^1^	16.0 ± 7.7	14.5 + 7.5	15.1 ± 7.8	14.5 ± 7.5
Boys only^1^	18.0 ± 7.3	16.8 ± 7.2	17.1 ± 7.6	16.5 ± 7.4
Girls only^1^	13.8 ± 7.4	12.1 ± 7.1	12.9 ± 7.4	12.5 ± 7.1
Stand and reach (cm)^1,3^	1.8 ± 6.5	1.5 ± 6.7	1.7 ± 6.7	1.1 ± 6.7
Boys only^1,3^	0.4 ± 6.4	-0.1 ± 6.5	0.0 ± 6.3	-0.6 ± 6.4
Girls only^1,3^	3.2 ± 6.4	3.1 ± 6.5	3.6 ± 6.6	2.8 ± 6.5
6-minute run (m)^1,2,3^	991 ± 133	979 ± 135	976 ± 134	961 ± 140
Boys only^1,2,3^	1023 ± 136	1012 ± 138	1006 ± 140	994 ± 146
Girls only^1,2,3^	957 ± 121	945 ± 122	944 ± 118	927 ± 125

*Note: Values are mean ± SD. ^1^2022 sig. different from 2016/17 after adjusting for age (p < 0.01); ^2^2022 sig. different from 2017/18 after adjusting for age (p < 0.01); ^3^2022 sig. different from 2018/19 after adjusting for age (p < 0.01).

Physical fitness also differed significantly across measurement years even after adjusting for age (Wilks' lambda = 0.72; p < 0.01) (Table 4). Specifically, performance on the countermovement jump (p < 0.001), agility run (p < 0.01), 6-minute run (p < 0.01) and stand-and-reach test (p ≤ 0.01) was lower in 2022 compared to the years prior to COVID-19. The medicine ball push performance, on the other hand, was significantly better post-COVID-19 compared to the previous years (p < 0.02).

**Figure 1.**
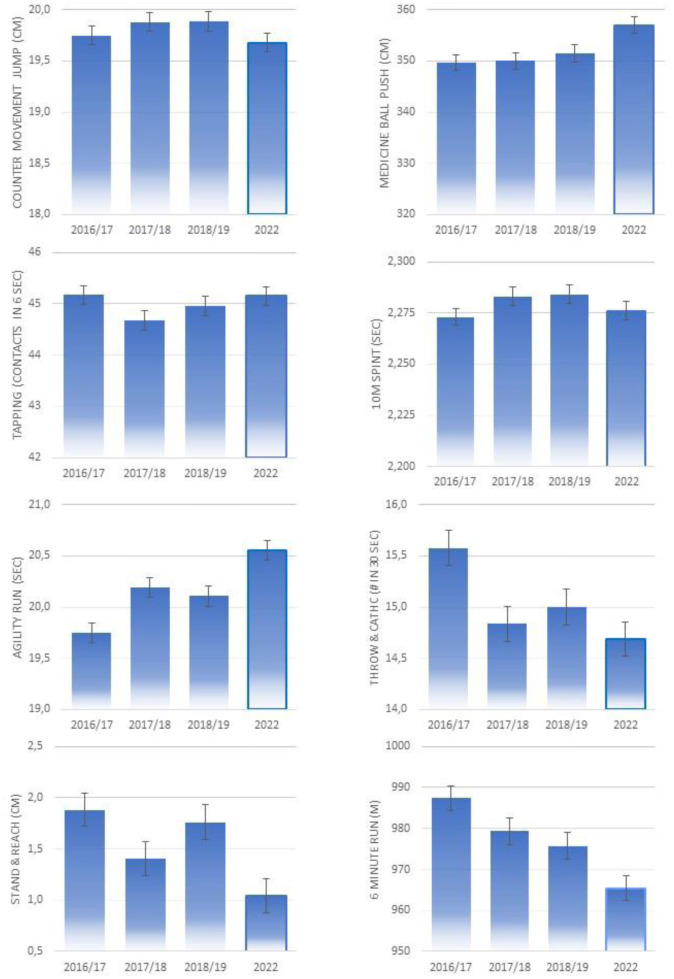
Components of physical fitness in the 2016/17, 2017/18 and 2018/19 school years and in 2022. Values are mean scores with 95% confidence intervals adjusted for age and BMIPCT.

In addition, participants performed worse at the throw-and-catch task in 2022 compared to 2016/17 (p < 0.01), but not as compared to the years of 2017/18 and 2018/19. Tapping performance, on the other hand, was better in 2022 compared to 2017/18 (p < 0.01), but not compared to the years of 2016/17 and 2018/19 after adjusting for age. No significant differences were observed for sprinting performance across the measurement years in the total sample. The results remained essentially unchanged after including BMIPCT as an additional covariate, except for the countermovement jump, where the performance was only significantly different between the years of 2022 and 2017/18, as well as 2018/19, respectively (Figure 1).

**Figure 2.**
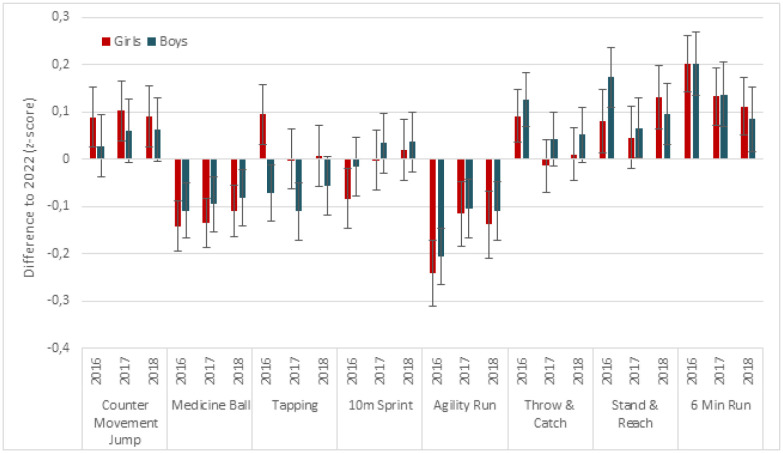
Difference in components of physical fitness (z-scores) between 2022 and previous years separately for girls and boys. Values are mean differences to 2022 with 95% confidence intervals adjusted for age.

Sex-specific analyses showed that girls performed significantly worse at the countermovement jump, agility run and the 6-min run in 2022 compared to the years prior to COVID-19 (p < 0.01) (Figure 2). The difference in flexibility was only significant between the years 2022 and 2016/17, as well as 2018/19, respectively (p < 0.01). There was no significant difference in countermovement jump performance in boys across the measurement years. Boys, however, displayed less flexibility in 2022, as indicated by the stand-and-reach test, as compared to all previous time points (p < 0.05), in addition to worse performance on the agility run and the 6-minute run (p < 0.01) (Figure 2). Performance on the medicine ball toss, on the other hand, was significantly better in boys and girls in 2022 compared to the years prior to COVID-19 (p < 0.01). Tapping performance was also significantly better in boys in 2022 as compared to the 2016/17 and 2017/18 school years (p ≤ 0.01), while there was no difference in the 10-m sprint. Girls, in contrast, displayed worse tapping and sprinting performance in 2022 as compared to 2016/17 (p < 0.01). Throw-and-catch performance was also significantly worse in 2022 as compared to 2016/17 in boys and girls (p < 0.01), but not when compared to the other measurement years.

After additionally adjusting for BMIPCT, the results remained essentially unchanged in boys. In girls, the results remained essentially unchanged except for the countermovement jump and sprinting performance. After adjusting for BMIPCT in addition to age, countermovement jump performance was only significantly better in 2017/18 compared to 2022 (p = 0.03), and sprinting performance no longer differed across measurement times.

## 4. Discussion

This study examined the effects of preventive measures to minimize the spread of COVID-19, which included social distancing, closure of schools and sports facilities over a 2-year period, on the physical fitness in Austrian elementary school children. The results showed a significantly higher BMIPCT in children post-COVID-19, with a more pronounced increase in girls. Further, cardiorespiratory endurance, agility and flexibility were significantly lower post-COVID-19 compared to the years preceding movement restrictions. The decline in linear speed and ball-handling skills in children, on the other hand, was already visible prior to the implementation of COVID-19 policies. Absolute strength, as indicated by the medicine ball push, on the other hand, was significantly higher after COVID-19 restrictions compared to the previous years. Sex-specific analyses showed that the decline in cardiorespiratory endurance and agility was comparable in boys and girls. Only girls, however, displayed consistently lower muscular power, while boys displayed consistently lower flexibility post-COVID-19 compared to the years prior to the implementation of COVID-19 policies.

The detrimental effects of COVID-19 policies on body weight have been shown in various studies across the globe [34],[42],[43],[50],[51]. Of particular concern is that weight gain was more pronounced in children who were already overweight or obese prior to the COVID-19 pandemic [34],[43],[50]. Previous research has also highlighted the importance of daily structure on body weight [52], which was significantly altered by the implemented policies. Due to school closures and a lack of access to extracurricular activities, including organized sports, obesogenic behaviors such as low PA, extensive screen time, a poor diet and poor sleep timing may have been exacerbated [36],[53]. Such behavioral changes, unfortunately, remained relatively stable after COVID-19 restrictions became less severe, which suggests that unfavorable effects of such policies are not spontaneously reversible [34],[54]. Rather, these behavioral patterns may reflect a new entrenched lifestyle that could exacerbate the current obesity epidemic. In addition to the well-established detrimental effects on chronic disease risk [55], excess body weight has also been associated with adverse outcomes in viral diseases such as COVID-19 [56],[57]. Accordingly, approaches to modify lifestyle changes that may have been required to limit the spread of the virus are urgently needed to address the unintended consequences of the implemented policies.

Given the impact of COVID-19 policies on children's lifestyle, it should not be surprising that physical fitness was affected as well. Even a few days of a sedentary lifestyle have been associated with physiological changes that were associated with decreased aerobic capacity and muscle loss [58]. In fact, physical inactivity has been shown to impair the oxidative capacity at all levels, including the cardiorespiratory system and oxidative function of skeletal muscle [58]. Accordingly, several studies showed a decline in cardiorespiratory endurance in children during the time when movement restrictions were implemented [45],[46],[59],[60]. Other studies, however, showed an increase in unstructured PA, such as playing outside, that may counteract the lack of access to organized sports [35],[38]. Engagement in habitual PA, however, also depends on the current fitness level, and it has been argued that children with higher fitness levels may be more resilient to policies that restrict access to structured PA [46],[61]. In addition, socioeconomic background and the living environment are critical correlates of PA and cardiorespiratory fitness, particularly in the absence of structured PA due to the closures of schools and recreational sports facilities [38],[40],[62]. Therefore, the impact of COVID-19 policies may have been stronger in children living in an urban environment with a lower socioeconomic background, who are already more vulnerable to lower fitness levels [63].

Consistent with the results of the present study, previous research showed a decline in flexibility [59] and agility [64], while muscular strength appears to be more resilient to negative effects of COVID-19 policies. Even though linear speed has been affected negatively by the implemented movement restrictions [64]–[66], the difference in linear speed between pre- and post-COVID-19 measurements in the present study was less pronounced than that observed for agility. The lower agility run performance, therefore, could also reflect a potential impact of COVID-19 policies on cognitive abilities. As this task requires memorizing specific forms of movement, working memory could affect performance as well, and there is evidence for lower executive functioning in elementary school children tested during the COVID-19 pandemic as compared to assessments before lockdowns were implemented [45]. Upper body strength, on the other hand, was higher in 2022, which is consistent with the results in German children [61]. Wahl-Alexander and Camic [47], on the other hand, reported a decline in push-up and sit-up performance. This decline may be attributed to the increase in body weight, which affects performance on exercises that need to move a person's body, while the present study evaluated absolute strength independent of body weight (i.e., medicine ball push). In this case, a higher body weight may actually contribute to better performance, as previous research showed better results at the medicine ball push in overweight children compared to their normal-weight peers [48]. The higher body weight, on the other hand, may have contributed to lower jumping performances post-COVID-19 as compared to pre-COVID-19 measurements [45],[67]. Other studies, however, showed better jumping performance post-COVID-19 despite detrimental changes in body weight [59],[62]. These conflicting data could suggest that there is a possibility to mitigate some negative effects of the COVID-19 pandemic in specific components of physical fitness via alternative activities, which include the implementation of online exercises and indoor workouts. Other aspects, such as cardiorespiratory endurance and agility, however, may require additional efforts to ensure sufficient engagement in physical activities that promote these components of physical fitness.

Some limitations of the present study also need to be considered when interpreting the results of the present study. There was no information on PA or participation in club sports, the socioeconomic background or the living environment of the participating children. Given the influence of household income and living situation (house vs. apartment), as well as access to a garden on PA levels during the pandemic [68]–[70], some children may have been able to cope with movement restrictions better than others. In addition, the implementation of online exercise programs most likely differed across schools. Because the same children were not measured over time, there may also be other confounding factors that could have affected the findings. The large sample size and the availability of multiple observations prior to the onset of the COVID-19 pandemic, on the other hand, are considerable strengths of this study, as they allow to differentiate between secular trends in physical fitness and the impact of COVID-19 policies.

## 5. Conclusions

In summary, the available data indicate that declines in physical fitness in children have been aggravated by the implementation of various policies that were intended to prevent the spread of the virus. As fitness during childhood is associated with PA and fitness in adulthood [71], the implemented policies could have significant unintended long-term consequences and impact clinical practice in years to come. While these results do not necessarily contradict the need for movement restrictions and social distancing that have been put into place, they highlight the importance of the development of public health actions and intervention strategies that reduce the negative impact of COVID-19 policies. Versatile opportunities for PA and innovative fitness programs that are anchored sustainably in schools and the community are needed to modify the currently unhealthy trajectories of physical fitness. Communities, policymakers and other relevant stakeholders also need to ensure access to opportunities for PA by providing access to environments that are conducive to and supportive of PA, particularly during times when the daily structure and access to organized sports is limited. In particular, elementary school children have been shown to unlikely be major contributors to the spread of COVID-19 [72] and school closures or restrictions to PA during school time, therefore, should be avoided in the future. The fact that individuals who engaged regularly in PA had an 11% lower risk of getting COVID-19 and a 34% lower risk of severe disease if they contracted the virus [73] further emphasizes the need to ensure adequate PA in order to mitigate the severity of future viral diseases.
